# Post-Concussion Syndrome and Functional Neurological Disorder: Diagnostic Interfaces, Risk Mechanisms, and the Functional Overlay Model

**DOI:** 10.3390/brainsci15070755

**Published:** 2025-07-16

**Authors:** Ioannis Mavroudis, Foivos Petridis, Eleni Karantali, Alin Ciobica, Sotirios Papagiannopoulos, Dimitrios Kazis

**Affiliations:** 1 Department of Neuroscience, Leeds Teaching Hospitals, Leeds LS9 7TF, UK; 2 Third Department of Neurology, Aristotle University of Thessaloniki, 54124 Thessaloniki, Greece; ubic2@otenet.gr (S.P.); dimitrios.kazis@gmail.com (D.K.); 3 School of Medicine, University of Leeds, Leeds LS2 9JT, UK; f_petridis83@yahoo.gr; 4 Department of Neurology, Democritus University of Thrace, 68100 Alexandroupolis, Greece; lena.kar@outlook.com; 5 Department of Biology, Faculty of Biology, Alexandru Ioan Cuza University of Iasi, Carol I Avenue 20th A, 700505 Iasi, Romania; alin.ciobica@uraic.ro; 6 Center of Biomedical Research, Romanian Academy, Iasi Branch, Teodor Codrescu 2, 700481 Iasi, Romania; 7 Academy of Romanian Scientists, 3 Ilfov, 050044 Bucharest, Romania; 8 Preclinical Department, Apollonia University, Păcurari Street 11, 700511 Iasi, Romania

**Keywords:** post-concussion syndrome, Functional Neurological Disorder, Functional Cognitive Disorder, mTBI, neuroticism, personality traits, trauma, cognitive symptoms, neuroimaging, functional overlay, diagnostic criteria, risk factors

## Abstract

Background: Post-concussion syndrome (PCS) and Functional Neurological Disorder (FND), including Functional Cognitive Disorder (FCD), are two frequently encountered but diagnostically complex conditions. While PCS is conceptualized as a sequela of mild traumatic brain injury (mTBI), FND/FCD encompasses symptoms incompatible with recognized neurological disease, often arising in the absence of structural brain damage. Yet, both conditions exhibit considerable clinical overlap—particularly in the domains of cognitive dysfunction, emotional dysregulation, and symptom persistence despite negative investigations. Objective: This review critically examines the shared and divergent features of PCS and FND/FCD. We explore their respective epidemiology, diagnostic criteria, and risk factors—including personality traits and trauma exposure—as well as emerging insights from neuroimaging and biomarkers. We propose the “Functional Overlay Model” as a clinical tool for navigating diagnostic ambiguity in patients with persistent post-injury symptoms. Results: PCS and FND/FCD frequently share features such as subjective cognitive complaints, fatigue, anxiety, and heightened somatic vigilance. High neuroticism, maladaptive coping, prior psychiatric history, and trauma exposure emerge as common risk factors. Neuroimaging studies show persistent network dysfunction in both PCS and FND, with overlapping disruption in fronto-limbic and default mode systems. The Functional Overlay Model helps to identify cases where functional symptomatology coexists with or replaces an initial organic insult—particularly in patients with incongruent symptoms and normal objective testing. Conclusions: PCS and FND/FCD should be conceptualized along a continuum of brain dysfunction, shaped by injury, psychology, and contextual factors. Early recognition of functional overlays and stratified psychological interventions may improve outcomes for patients with persistent, medically unexplained symptoms after head trauma. This review introduces the Functional Overlay Model as a novel framework to enhance diagnostic clarity and therapeutic planning in patients presenting with persistent post-injury symptoms.

## 1. Introduction

Post-concussion syndrome (PCS) is a recognized clinical outcome of traumatic brain injury (TBI), presenting as a constellation of symptoms that may include headache, dizziness, mood and anxiety disturbances, and cognitive impairments [1]. Although the designation “post-concussion syndrome” dates back to 1934 [2], the term “post-TBI syndrome” is sometimes preferred, as similar symptom profiles can emerge not only after concussive events but also following moderate, severe, or even sub-concussive head trauma [1]. Considerable debate surrounds PCS, particularly in cases where symptoms persist beyond the expected recovery period [3]. Persistent PCS is often marked by subtle and subjective complaints, lacking clearly defined neuropathological correlates. Its symptoms are not specific to brain trauma and are commonly observed in the general population, complicating diagnostic clarity and potentially leading to underrecognition in clinical practice. Moreover, standard neuropsychological assessments used in the chronic phase frequently fail to detect objective deficits, despite patients reporting significant cognitive disturbances [4].

The population affected by PCS is heterogeneous, encompassing individuals with varying injury severity, mechanism, and psychosocial context. This variability suggests that individual factors, such as pre-injury psychological profile or stress sensitivity, may play a critical role in shaping post-injury symptom expression [1]. Reported prevalence rates of PCS following mild TBI vary considerably—from 30% to as high as 80% [5]—largely reflecting inconsistencies in diagnostic definitions and study populations. While early investigations attempted to link PCS risk to injury severity using tools like the Glasgow Coma Scale (GCS), duration of unconsciousness, or neuroimaging findings [6,7,8,9,10], such metrics have proven unreliable in predicting long-term outcomes. Instead, emerging evidence indicates that prior concussions—especially when recent or recurrent—may heighten the risk of prolonged symptomatology following subsequent head trauma [11].

Functional Neurological Disorder (FND) is increasingly recognized as a prevalent and burdensome neuropsychiatric condition, characterized by motor, sensory, or cognitive symptoms that are incompatible with recognized neurological disease. Once considered rare or psychogenic, FND is now understood to be a leading cause of disability in outpatient neurology and accounts for a substantial share of tertiary care referrals [12].

Finkelstein et al. estimate the point prevalence of FND between 4 and 50 per 100,000 persons depending on methodological rigor and diagnostic criteria [12]. FND comprises 6–16% of new consultations in neurology outpatient settings, with motor symptoms such as tremor, limb weakness, and gait disturbance being most common. In epilepsy units, up to one-third of admitted patients ultimately receive a diagnosis of psychogenic non-epileptic seizures (PNES). Annual incidence estimates for PNES alone range from 1.4 to 4.9 per 100,000 [13,14,15,16,17,18,19,20,21].

Subtype data underscore the variability of FND presentations. Functional motor symptoms show an incidence of 1.5–4.0 per 100,000 person-years, while functional cognitive disorder (FCD), though underrepresented in general epidemiological studies, is highly prevalent in memory clinics and younger patients with subjective cognitive complaints but no evidence of neurodegeneration [12,22]. FCD also overlaps with somatic symptom burden and attentional dysfunction, complicating diagnostic clarity.

FND disproportionately affects populations with psychiatric comorbidity, trauma history, and socioeconomic disadvantage. Studies cited in the review highlight elevated rates in refugee populations, military veterans, and individuals exposed to interpersonal violence [23,24,25]. These findings support a biopsychosocial model of vulnerability, where cumulative stress and emotional dysregulation modulate the expression of functional symptoms.

A critical barrier to care remains diagnostic delay, often spanning several years between symptom onset and formal recognition. Misdiagnosis as epilepsy, chronic fatigue syndrome, or even malingering is common, especially in patients presenting with subtle or fluctuating symptoms [26,27].

## 2. Diagnostic Criteria: PCS vs. FND and Functional Cognitive Disorder

### 2.1. Post-Concussion Syndrome (PCS)

PCS, as previously defined in ICD-10 (F07.2), represents a constellation of symptoms—such as headache, dizziness, fatigue, sleep disturbance, irritability, and concentration or memory difficulties—emerging within days to weeks after a concussion or mild TBI [28]. However, ICD-11 removed PCS as a discrete diagnosis due to its nonspecific clinical utility, recommending instead the term “persisting concussion symptoms” or categorization under neurocognitive disorders due to TBI [29]. Similarly, the DSM-5 does not list PCS; instead, a subset of these symptoms may be classified as Mild Neurocognitive Disorder Due to Traumatic Brain Injury, which requires objective evidence of cognitive decline in one or more domains, based on neuropsychological assessment [30].

In practice, PCS remains a symptom-based label rather than a distinct neuropathology. Its diagnosis relies heavily on clinical history and subjective reporting, often without corroborating cognitive deficits on testing. This disparity illustrates a key challenge: while many patients report ongoing complaints, few meet criteria for a formal neurocognitive disorder.

### 2.2. Functional Neurological Disorder (FND)

Unlike PCS, FND is recognized in both ICD-11 (6C20) and DSM-5 (300.11) based on positive clinical findings—not merely the exclusion of organic pathology [29]. Core DSM-5 criteria include [30]:

1. One or more symptoms of altered voluntary motor or sensory function;
2. Clinical evidence showing incompatibility between symptoms and recognized neurological disease;
3. Symptoms not better explained by another medical or mental disorder;
4. Symptoms causing significant distress or impairment.

Key positive signs in motor FND include Hoover’s sign (normal hip extension during contralateral flexion), tremor entrainment/distractibility, inconsistent gait patterns, and sensory inconsistencies. In non-epileptic seizures, features such as prolonged unresponsiveness, closed eyes during episodes, and asynchronous limb movements serve as positive indicators.

### 2.3. Functional Cognitive Disorder (FCD)

FCD falls within the FND spectrum, characterized by persistent cognitive complaints—forgetfulness and concentration issues—despite normal or inconsistent objective cognitive performance. Positive diagnostic features include:

• Performance variability: Good real-world functioning but poor test consistency;
• Metacognitive distortion: Over-focusing on minor lapses;
• Symptom improvement with distraction: e.g., fluency on testing improves when anxiety is redirected.

Differentiating FCD from neurodegenerative disorders relies on preserved functionality, absence of objective deficits, and demonstration of positive incongruence during testing.

### 2.4. Cognitive Profiles: FCD vs. PCS

Although both PCS and FCD present with cognitive complaints such as memory lapses and attentional difficulty, their neurocognitive patterns diverge significantly. In PCS, objective testing may reveal subtle inefficiencies in memory encoding, processing speed, or divided attention. By contrast, FCD is characterized by performance variability, over-monitoring, and incongruence between complaint and capacity. For example, patients with FCD may demonstrate preserved narrative fluency despite self-reported verbal memory impairment or show improvement with distraction during testing. Table 1 highlights these distinctions. Recognizing these cognitive fingerprints is essential for validating functional contributions in chronic PCS cases.

### 2.5. Comparison and Clinical Implications

While PCS is best understood as a broad symptom label rather than a discrete nosological entity, FND (including FCD) is diagnosed using positive clinical criteria and examination findings. This distinction allows earlier recognition and more precise treatment targeting mental and cognitive symptomatology rooted in functional pathophysiology rather than post-injury sequelae.

## 3. Personality Traits and Risk Factors

### 3.1. Post-Concussion Syndrome

Post-Concussion Syndrome (PCS) is increasingly recognized not only as a physiological aftermath of mild traumatic brain injury (mTBI), but as a condition deeply intertwined with psychological, behavioral, and personality factors. While traditional models emphasized direct mechanical injury and assumed spontaneous resolution, contemporary evidence underscores a more complex interaction between predisposing personality traits, psychosocial vulnerabilities, subjective symptom reporting, and neurobiological plasticity in shaping the clinical evolution of PCS [31].

Among the most studied and consistently reported psychological correlates of prolonged PCS is the trait of neuroticism. Atif et al. (2022) [32] found that adolescents with persistent PCS exhibited significantly elevated levels of neuroticism, along with reduced extraversion, agreeableness, and conscientiousness, when compared to healthy controls. This personality profile is marked by emotional instability, hypersensitivity to stress, and a propensity toward negative affective states, all of which may amplify the perception and reporting of post-concussive symptoms. These adolescents also displayed lower emotional resilience and diminished coping capacity, factors that may further erode the ability to adapt to post-injury stressors and physiological changes.

Wood et al. (2019) [33] emphasized that trait anxiety, alexithymia, and depressive tendencies were powerful predictors of prolonged PCS. In their cohort, the combination of difficulty identifying and expressing emotions (alexithymia), together with heightened anxiety sensitivity, explained a substantial portion of the variance in PCS symptom intensity. Such traits appear to act as amplifiers, heightening bodily awareness and symptom vigilance while simultaneously reducing adaptive emotional processing capacity [33].

The emotional profiles of PCS patients are often marked by somatic trait anxiety, which refers to the habitual tendency to interpret physical sensations as signs of serious illness. This pattern is common in patients with lingering PCS, who frequently endorse somatic symptoms such as fatigue, dizziness, and headaches in the absence of overt objective pathology [34]. These symptoms are not merely malingering or exaggeration but rather may emerge from maladaptive interoceptive processing shaped by long-standing psychological predispositions.

Further compounding the picture is the observation that subjective cognitive complaints, one of the most prevalent features of PCS, often bear little correlation to objective neuropsychological performance. Both Atif et al. and Wood et al. noted that participants routinely reported memory lapses, attentional difficulties, and “brain fog,” yet they performed normally on standardized tests of cognitive function [32,33]. This dissociation suggests that cognitive symptoms in PCS may be better understood through the lens of perception and belief rather than cortical dysfunction per se. Such discrepancies are often maintained by attentional biases and catastrophic interpretations of benign cognitive errors. Patients may misattribute ordinary lapses—such as forgetting a word or misplacing an object—as evidence of irreversible brain injury, particularly when such experiences follow an emotionally distressing event like a concussion. In individuals high in neuroticism or low in psychological flexibility, these cognitive misattributions may become self-reinforcing, feeding a cycle of fear, avoidance, and heightened symptom monitoring [34].

The risk of persistent PCS is not uniform across populations. Recent data consistently indicate that certain demographic and psychological factors heighten vulnerability to chronic symptoms. Female sex, a history of psychiatric illness, low to medium levels of education, poor sleep quality prior to injury, and pre-existing pain conditions have all been associated with prolonged symptom burden [34,35]. Of these, the presence of a prior psychiatric diagnosis—particularly mood and anxiety disorders—was a particularly robust predictor of symptom chronicity [35].

Recovery trajectories following mTBI are heterogeneous. King et al. used longitudinal modeling to identify four distinct symptom trajectories among their participants. While most individuals either recovered quickly or improved gradually, approximately 13% of the cohort demonstrated a deteriorating trajectory, marked by symptom worsening at the six-month follow-up [35]. This group was disproportionately composed of individuals with elevated pre-injury psychological distress, greater initial symptom burden, and higher levels of cognitive-affective interference.

These findings lend support to the Fear Avoidance Model (FAM) of symptom maintenance, in which individuals with high anxiety sensitivity and a tendency to catastrophize bodily sensations avoid cognitively or physically demanding activities. This avoidance leads to deconditioning, social withdrawal, and increased time spent ruminating on symptoms, which in turn reinforces the belief in having sustained serious neurological damage. Recent studies demonstrated that pain catastrophizing and cognitive distortions regarding symptom permanence were strong predictors of delayed recovery, even when injury severity was mild [32,36,37].

Taken together, these studies paint a cohesive picture in which persistent PCS is often less about the extent of mechanical injury and more about how individuals perceive, interpret, and emotionally respond to their post-injury state. The data strongly suggest that PCS may, in many cases, represent a complex neuropsychiatric syndrome in which brain injury interacts with individual psychological makeup to produce enduring and disabling symptoms.

Recognizing this interplay has important clinical implications. Early screening for personality traits such as neuroticism, somatic anxiety, and alexithymia may help to identify those at greatest risk of prolonged symptoms. Interventions targeting these psychological domains—particularly through cognitive behavioral therapy, psychoeducation, and resilience training—may interrupt the feedback loops that sustain chronic PCS. In the context of normal neuroimaging and unremarkable neuropsychological assessments, clinicians should maintain a high index of suspicion for the influence of maladaptive cognitive and emotional patterns in perpetuating symptom complaints.

Ultimately, the emerging consensus is that post-concussive symptoms cannot be viewed solely as the sequelae of biomechanical trauma. They are best understood as biopsychosocial phenomena shaped by pre-injury traits, post-injury beliefs, and the dynamic interactions between the brain, behavior, and the environment. Addressing these dimensions is not ancillary but central to improving outcomes for patients with persistent PCS.

### 3.2. Personality Traits and Psychological Profiles in Functional Neurological Disorder (FND) and Functional Cognitive Disorder (FCD)

The role of personality traits in Functional Neurological Disorder (FND), including Functional Cognitive Disorder (FCD), has been increasingly examined as part of a broader biopsychosocial understanding of symptom emergence and maintenance. Rather than reflecting fixed causal determinants, personality characteristics appear to shape symptom interpretation, emotional regulation, interoceptive focus, and therapeutic response.

Among the most consistently reported personality features in FND is neuroticism, a trait denoting heightened emotional reactivity, vulnerability to stress, and a predisposition toward anxiety and worry. Stone et al. (2020) found that patients with functional limb weakness scored significantly higher in neuroticism compared to both healthy and neurological controls, with this trait correlating with predisposing psychological factors such as anxiety and prior adversity [38]. This elevated neuroticism has been echoed in FCD populations, where it is also associated with excessive monitoring of cognitive performance and catastrophic interpretations of benign lapses [39].

In addition to neuroticism, conscientiousness emerged as a distinguishing trait, particularly in patients with FCD. De Vroege and colleagues (2022) observed that FCD patients scored higher in this domain compared to both psychiatric and neurological cognitive disorder controls [39]. While typically adaptive, high conscientiousness in this population may reflect a form of perfectionism that fosters hyper-attention to cognitive details, reduced tolerance for errors, and persistent rumination on perceived inadequacies. When combined with elevated neuroticism, this trait profile may produce a vulnerability to developing functional symptoms rooted in misinterpreted internal cues and performance concerns.

Another trait of interest is openness to experience, particularly its cognitive facets such as intellectual curiosity and flexibility. De Vroege et al. (2022) found that openness was elevated in FCD patients, especially in the “ideas” facet of the NEO-PI-R [39]. This trait may underlie greater metacognitive focus and introspective tendencies, which could enhance symptom awareness and sensitivity to subtle fluctuations in mental performance. However, Stone et al. (2020) conversely noted that patients with functional limb weakness showed reduced openness, suggesting that differences in symptom subtype may influence the expression and impact of personality traits [38]. Lower openness may relate to greater rigidity in symptom interpretation and resistance to psychological explanations.

Pun et al. (2020), in a large retrospective audit of 288 patients from an Australian multidisciplinary FND clinic, reinforced the clinical relevance of these personality profiles [40]. Their findings highlighted the psychological heterogeneity within FND, categorizing patients into at least three broad subtypes: an “anxious substrate” group dominated by somatic vigilance and trait anxiety; a “trauma-dissociative” group characterized by early adverse experiences and impaired emotional integration; and a “neurodevelopmental” group including individuals with comorbid ASD, ADHD, or intellectual disabilities. These groupings reflect the diverse ways in which personality traits, trauma, and developmental history may converge to influence FND symptomatology.

The relationship between trauma and personality traits further deepens this complexity. Stone et al. (2020) reported that childhood trauma, particularly emotional neglect and abuse, was significantly more prevalent among FND patients than controls [38]. These adverse experiences are known to potentiate high neuroticism and emotional dysregulation, and may mediate maladaptive coping strategies such as dissociation, hypervigilance, or cognitive avoidance. In this context, personality is not a static backdrop but part of a dynamic interaction between early experiences, emotional regulation capacity, and neural plasticity.

Emerging cognitive models of FND, including Bayesian brain frameworks, suggest that maladaptive personality traits may amplify the influence of prior beliefs and expectations on sensory experience. Individuals high in neuroticism may possess overly precise prior expectations about illness or cognitive failure, which in turn dominate ambiguous sensory input and produce functionally disabling symptoms [41]. Similarly, those with high conscientiousness and perfectionism may continually test and monitor their performance, feeding into cycles of uncertainty and symptom amplification [39].

These trait-driven cognitive-emotional loops are not merely theoretical. Pun et al. (2020) described how dissociative FND patients with neurodevelopmental histories often exhibited features consistent with impaired metacognition, low emotional granularity, and reduced psychological insight—all of which are relevant to therapeutic planning [40]. Patients in this subgroup were more likely to resist conventional explanatory models, require multimodal intervention, and exhibit slower recovery trajectories.

Clinically, these findings support a stratified care approach in which personality assessment becomes a core component of diagnostic formulation. Personality-informed treatment can allow for tailored therapeutic strategies—such as metacognitive therapy for perfectionism and cognitive misattribution, emotion regulation training for high neuroticism, and psychoeducation for individuals with low openness. Recognizing these traits also helps to anticipate therapeutic alliance challenges and informs the pacing and framing of psychological interventions.

Ultimately, personality traits do not cause FND, but they contribute to a fertile context in which symptoms may arise and be sustained. Integrating trait assessment into clinical care allows for a deeper appreciation of individual vulnerability, symptom meaning, and the nuanced psychological terrain that characterizes functional disorders.

### 3.3. Risk Factors in Functional Neurological Disorder and Post-Concussion Syndrome: A Comparative Perspective

Understanding the etiological architecture of both Functional Neurological Disorder (FND) and Post-Concussion Syndrome (PCS) necessitates an exploration of the complex interplay of biological, psychological, and social factors. Both conditions, though arising from seemingly different origins—FND from functional disturbances in the nervous system and PCS from mild traumatic brain injuries (mTBI)—share overlapping risk mechanisms. These include early trauma, neurodevelopmental vulnerabilities, maladaptive personality traits, and sociocultural influences that perpetuate symptom chronicity.

## 4. Predisposing, Precipitating and Perpetuating Factors

### 4.1. Predisposing Factors

The most consistent predisposing factors in FND involve a history of childhood trauma—physical, sexual, or emotional abuse—with meta-analyses confirming its overrepresentation, particularly in functional seizures (PNES). Neurodevelopmental traits such as autistic spectrum characteristics and ADHD symptoms are prevalent in patients with Functional Cognitive Disorder (FCD), possibly reflecting sensory processing anomalies and predictive coding impairments [38,39,40].

Psychiatric comorbidity, especially depression and anxiety, is commonly documented, although notably about one-third of patients have no formal psychiatric diagnosis, emphasizing that while these factors contribute to risk, they are not essential for disease manifestation.

In PCS, female sex emerges as a consistent predisposing factor, with numerous studies reporting prolonged recovery times and more severe symptom burdens among women. Age also plays a nuanced role; middle-aged individuals (35–49 years) and older adults demonstrate a higher risk for persistent symptoms, possibly due to diminished neuroplasticity or age-related comorbidities [33,34].

A history of mental health disorders—especially anxiety, depression, and attention-deficit disorders—significantly increases the risk for prolonged symptoms. Somatization, characterized by the propensity to experience and communicate somatic distress in response to psychological stress, is another crucial predictor, especially in females [34].

### 4.2. Precipitating Factors

FND symptoms often follow acute stressors such as grief, interpersonal conflict, or significant life transitions. In physical terms, seemingly minor injuries, including surgical interventions or infections, frequently precede symptom onset. The phenomenon of iatrogenic harm—where vague diagnostic language or excessive testing without explanatory clarity—can itself become a trigger by increasing uncertainty and fear [38,39,40].

In PCS, the precipitating factor is typically a mild TBI sustained during sports, vehicular accidents, or falls. Yet, intriguingly, the severity of the initial injury, as measured by conventional neuroimaging or Glasgow Coma Scale scores, does not reliably predict PCS. Instead, early symptom burden—such as acute headaches, dizziness, or nausea—is a more robust predictor of prolonged symptoms [32,34].

Previous concussions—particularly multiple events within a short time frame—further compound the risk, suggesting that cumulative brain trauma sensitizes neural and cognitive systems to dysfunction [42].

### 4.3. Perpetuating Factors

Chronicity in FND is sustained by cognitive-behavioral loops: hypervigilance to bodily symptoms, catastrophic misinterpretations, and avoidance behaviors reinforce the functional disturbance [38]. Social factors, including reinforcement by family, avoidance of responsibilities, and even litigation, can unintentionally solidify illness identity. Crucially, how healthcare professionals communicate the diagnosis can alter the trajectory of the illness; positive, clear, and confident explanations reduce distress and improve outcomes [38,39,40].

In PCS, involvement in legal proceedings—often due to injury claims—has been strongly correlated with a wider range and longer duration of symptoms, highlighting the role of psychosocial and external stressors in symptom perpetuation [33,34]. Additionally, low physical activity levels in the subacute period post-injury are associated with poorer recovery, countering earlier recommendations for strict rest [43].

Social isolation and low levels of perceived support, particularly in adolescents, also increase the risk of persistent symptoms, pointing to the necessity of integrative biopsychosocial rehabilitation models [44].

### 4.4. Shared Personality Profiles

Across both disorders, personality traits such as high neuroticism, harm avoidance, and perfectionism have been documented. In FND, particularly FCD, these traits may amplify attentional biases toward bodily dysfunction, contributing to the subjective experience of cognitive decline despite preserved objective function. In PCS, neuroticism and negative emotionality also correlate with higher symptom severity and delayed recovery [33,34,38,39].

### 4.5. Converging Pathophysiological Considerations

**Figure 1:**
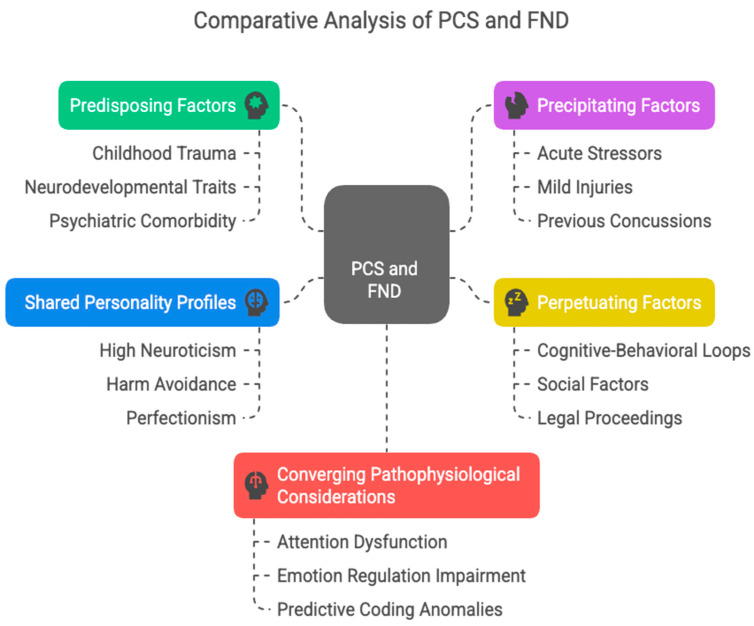
Comparative biopsychosocial model of shared mechanisms in Post-Concussion Syndrome (PCS) and Functional Neurological Disorder (FND). This figure outlines key overlapping domains between PCS and FND, structured around a biopsychosocial framework. The model includes predisposing factors (green): childhood trauma, neurodevelopmental traits, and psychiatric comorbidity that establish vulnerability; precipitating factors (purple): stressors, mild injuries, or prior concussions that may initiate symptom expression; perpetuating factors (yellow): psychosocial and cognitive mechanisms that maintain chronic symptoms, including behavioral loops and legal stressors; shared personality profiles (blue): traits such as high neuroticism, harm avoidance, and perfectionism that enhance symptom monitoring and catastrophizing; and converging pathophysiological considerations (red): network-level dysfunctions involving attention, emotional regulation, and predictive coding, seen across both conditions in neuroimaging studies.

Although PCS is typically triggered by biomechanical injury and FND by psychological or idiopathic origins, recent neuroimaging and neurobiological studies suggest overlapping dysfunctions in attention, emotion regulation, and predictive coding (Figure 1, Table 2). The converging evidence positions both disorders within a broader framework of brain-body integration breakdown, underscoring the importance of personalized treatment plans based not only on the mode of symptom onset but also on the patient’s psychosocial context and personality architecture [34]. While this review focuses on mild TBI and functional disorders, it is worth noting that some patients with severe TBI who are non-verbal or minimally conscious may also exhibit subtle signs of cognitive awareness.

## 5. Neuroimaging in Post-Concussion Syndrome and Functional Neurological Disorder

### 5.1. Post-Concussion Syndrome

Despite PCS being considered a “mild” traumatic brain injury sequela, extensive neuroimaging studies challenge the notion that the brain fully recovers from such injuries. Echlin et al. (2021) synthesized findings from 34 neuroimaging studies conducted a decade or more after mild TBI or repetitive head injuries (RHI), illustrating a range of structural and functional alterations in various populations, including athletes, veterans, and the general public [45].

One of the most replicated findings involves abnormalities in white matter microstructure, as evidenced by diffusion tensor imaging (DTI). Multiple studies cited in the review report reduced fractional anisotropy (FA) and increased radial and axial diffusivity in key white matter tracts, such as the corpus callosum, superior longitudinal fasciculus, and uncinate fasciculus [45]. These disruptions suggest long-standing microstructural damage, particularly in retired athletes exposed to concussions and RHIs over the years.

Additionally, cortical thinning has been observed in frontal, parietal, and temporal regions, areas critical to cognition and emotional regulation. Notably, the hippocampus and amygdala—regions involved in memory and emotional processing—were consistently reported to be smaller in those with histories of mTBI or RHI exposure [45].

Functional MRI (fMRI) and magnetoencephalography (MEG) have further revealed hypoconnectivity and compensatory hyperactivation in the prefrontal cortex, posterior cingulate, and default mode network nodes. These patterns suggest that even in asymptomatic individuals, lasting changes in brain connectivity persist, potentially explaining the subjective cognitive complaints typical of PCS [46].

Studies employing positron emission tomography (PET) have uncovered increased tau deposition in cortical and subcortical regions of retired athletes, raising concerns about neurodegenerative potential in PCS-like states, although clinical implications remain debated [47,48].

Proton magnetic resonance spectroscopy (MRS) has shown increased myo-inositol and choline levels, both markers of glial activation and neuroinflammation, even decades after the initial injury. Similarly, altered cerebral blood flow (CBF) has been detected via arterial spin labeling and perfusion-weighted imaging, with specific reductions in the temporal poles and elevations in parietal regions—anomalies that correlate with poorer verbal memory and naming abilities [49].

Collectively, these findings reinforce that PCS may not always be “mild” in its aftermath and can involve diverse, lasting neurobiological changes detectable with sensitive imaging modalities.

### 5.2. FND: Disrupted Networks and Functional Correlates

While PCS shows structural and metabolic brain alterations, Functional Neurological Disorder (FND)—long characterized as a “non-organic” condition—is now increasingly understood through a network-based functional neuroimaging lens. Perez et al. (2021) presented compelling neuroimaging findings that support the presence of measurable brain abnormalities in FND [50].

Across multiple fMRI studies, FND patients show altered activity and connectivity in brain regions implicated in emotion regulation, self-agency, and interoception. These include the anterior cingulate cortex (ACC), insula, amygdala, and supplementary motor area (SMA). Notably, emotion–motor interface circuits show hyperconnectivity with limbic structures, reinforcing the model of “emotion-driven movement disorders” in FND [50].

In individuals with Functional Cognitive Disorder (FCD), abnormal activation of the default mode network (DMN) and frontoparietal attention systems has been reported. These patients often demonstrate reduced deactivation of the DMN, which may explain persistent self-monitoring and preoccupation with cognitive failures—central symptoms in FCD [50].

Resting-state studies indicate network-level dysfunction rather than focal pathology. Aberrant connectivity between the posterior cingulate cortex, prefrontal cortex, and sensorimotor systems point to impaired integration of sensory input with motor output and a disrupted sense of agency, particularly relevant in functional motor symptoms [51,52].

Interestingly, the prefrontal cortex, limbic areas, and parietal regions implicated in FND overlap with the regions affected in PCS, particularly when persistent subjective cognitive complaints are present. Both disorders show disrupted functional circuits involved in attention, emotion, and executive control, suggesting shared pathophysiological substrates in some cases—despite differing etiologies and clinical narratives.

Although FND and PCS originate from distinct clinical frameworks—functional versus organic—neuroimaging evidence reveals overlapping vulnerabilities in fronto-limbic circuits, default mode network dysregulation, and white matter connectivity. These findings offer a neurobiological explanation for why post-concussion symptoms may mirror or overlap with functional symptoms, particularly in cases of persistent, medically unexplained complaints. Crucially, these insights advocate for integrative diagnostic models that move beyond binary organic vs. non-organic classifications and toward an appreciation of brain network dysfunction as a shared terrain in both conditions.

## 6. Biomarkers and Diagnostic Differentiation in PCS and FND

Traditional protein biomarkers, including S100B, GFAP, UCH-L1, neurofilament light chain (NF-L), and tau, were initially markers for acute brain injury, but their role in chronic PCS is less clear due to inconsistent long-term elevation and limited correlation with symptom severity [53]. Persistent neurological deficits and protracted symptoms have been associated with sustained elevation of GFAP and NF-L, although results across studies are not always concordant. This inconsistency can be explained by temporal and methodological heterogeneity. GFAP and NF-L levels may spike in the acute post-injury phase (hours to days) but normalize despite persistent symptoms. Emerging biomarker candidates such as microRNAs and extracellular vesicles show promise for specificity in chronic PCS, transcending the limitations of traditional protein markers (Table 3).

A multi-modal biomarker approach is beginning to reveal systemic and non-invasive indicators of FND. Cortisol levels in saliva or serum have been proposed as markers of symptom state, trait vulnerability, and prognosis [54]. In Functional Neurological Disorder (FND), the presence of positive clinical signs—particularly network-level functional disturbances identified through advanced neuroimaging—offers objective diagnostic support, shifting the diagnostic approach away from exclusion and enhancing clinical confidence. By contrast, while Post-Concussion Syndrome (PCS) benefits from established acute-phase biomarkers, the challenge lies in differentiating PCS from FND or other post-injury syndromes over time. In this context, longitudinal evaluations that incorporate metabolic imaging and inflammatory biomarker panels hold promise in distinguishing persistent functional symptoms from trauma-induced neurological changes.

Both PCS and FND have advanced well beyond purely subjective definitions. Biomarkers spanning neural, systemic, and functional domains offer a pathway to more precise diagnosis, etiological understanding, and targeted treatment. The future lies in integrating neuroimaging, blood-based markers, and functional measures to clarify clinical boundaries and guide tailored intervention.

## 7. Discussion

Although Post-Concussion Syndrome (PCS) and Functional Neurological Disorder (FND), including its cognitive subtype, Functional Cognitive Disorder (FCD), are often perceived through different nosological lenses, clinical and neurobiological observations suggest a more nuanced relationship. These conditions may share phenotypic expressions, such as subjective cognitive complaints, fatigue, and emotional dysregulation, yet arise from fundamentally different etiologies and pathophysiological mechanisms. Distinguishing between them, particularly in cases of persistent symptoms post mild traumatic brain injury (mTBI), is essential for appropriate diagnosis, management, and prognosis.

PCS is characterized by a constellation of symptoms following mechanical brain injury, typically mild TBI or concussion. Its core features include headache, dizziness, concentration difficulties, memory lapses, irritability, sleep disturbance, and fatigue. Critically, PCS has a defined initiating event—a biomechanical insult to the brain—providing an objective, time-anchored cause. Even when standard imaging appears normal, advanced neuroimaging has consistently revealed microstructural disruptions, altered functional connectivity, metabolic abnormalities, and neuroinflammatory signatures that persist beyond the acute phase. These findings support PCS as an organic post-traumatic condition rather than a purely psychological or functional disorder.

While PCS symptoms often resolve within weeks to months, a subset of patients report persistent complaints lasting months or even years. The persistence of symptoms does not, by itself, challenge the organic origin of PCS; however, clinical vigilance is required when presentation deviates from expected trajectories.

By contrast, FND and FCD are functional disorders, defined not by structural brain damage but by the altered functioning of brain networks involved in attention, agency, emotion, and interoception. The hallmark of these disorders lies not in the absence of symptoms but in symptom incongruity, inconsistency, and reversibility under examination, paired with positive diagnostic signs such as Hoover’s sign (in motor FND) or dissociation between subjective complaint and objective test performance (in FCD).

In FCD, patients often report difficulties with memory, concentration, or word-finding—sometimes indistinguishable from PCS complaints—but neuropsychological testing typically reveals intact performance, with patterns of over-monitoring, attentional dysregulation, and disproportionate symptom focus. These features contrast with the subtle but often demonstrable cognitive deficits in PCS, particularly in memory encoding, divided attention, and processing speed domains. Post-Concussion Syndrome (PCS) and Functional Neurological Disorder/Functional Cognitive Disorder (FND/FCD) share several clinical characteristics despite differing etiologies. Both conditions are frequently marked by subjective cognitive complaints that appear disproportionate to objective neuropsychological findings, emotional symptoms such as anxiety, depression, and irritability, as well as fatigue, sleep disturbances, and heightened somatic vigilance often accompanied by symptom catastrophizing. Moreover, overlapping risk factors, including female sex, a personal or family history of psychiatric illness, elevated neuroticism, prior trauma exposure, and maladaptive coping mechanisms, are common across both populations. These shared psychosocial and behavioral vulnerabilities contribute to diagnostic ambiguity, particularly in chronic post-injury presentations where symptoms no longer correlate clearly with the initial mechanical trauma.

While Post-Concussion Syndrome (PCS) and Functional Neurological Disorder/Functional Cognitive Disorder (FND/FCD) may present with overlapping clinical features, several red flags should prompt careful consideration of a functional diagnosis. Key indicators include clear inconsistencies between the reported mechanism of injury and the severity of symptoms—such as disabling cognitive complaints following a low-impact trauma—as well as subjective impairments that greatly exceed objective neuropsychological findings, particularly when cognitive testing reveals intact or even above-average performance. Discrepancies across cognitive domains, like severe verbal memory deficits in individuals who nonetheless demonstrate fluent, coherent narrative recall, further underscore functional involvement. Positive functional signs, including distractibility of symptoms, fluctuating performance, or paradoxical improvements under dual-task conditions, serve as critical diagnostic clues. In cases exhibiting such features, particularly when contextualized within a psychosocial risk framework, including prior psychiatric illness, trauma, or personality vulnerabilities, FND or FCD should be considered either as the primary diagnosis or as a functional overlay superimposed on an initial organic insult. Importantly, the clinical aim is not to establish a binary between “real” (PCS) and “functional” (FND/FCD) conditions but rather to adopt a continuum-based view of brain dysfunction. This model acknowledges that some patients may transition from an acute, mechanically triggered syndrome to one sustained by functional mechanisms. In such cases, integrative care is essential. This involves early identification of psychological vulnerabilities and maladaptive illness beliefs, vigilant monitoring for functional symptom patterns, and collaboration across neurology, psychiatry, psychology, and rehabilitation. Diagnostic flexibility and clinical humility are vital, allowing for iterative re-evaluation as symptom trajectories evolve over time.

The Functional Overlay Model provides a clinically useful framework for understanding and managing cases where symptoms persist beyond the expected physiological recovery following mild traumatic brain injury (mTBI), particularly when investigations yield normal or non-specific findings, including neuroimaging and detailed neuropsychological assessments [31]. This model becomes especially pertinent in cases characterized by clinical inconsistencies, such as a marked discrepancy between subjective complaints and objective evidence, or when symptoms appear exaggerated, fluctuating, or incongruent with the known biomechanical impact of the injury. In these scenarios, the model guides clinicians toward identifying functional mechanisms superimposed on the initial organic insult. These mechanisms often arise in the context of predisposing factors—such as psychological distress, maladaptive beliefs, trauma history, or personality traits—and are perpetuated by secondary gains, heightened symptom vigilance, and illness-related expectations. Recognizing and intervening on these maintaining factors early in the course of the illness facilitates more accurate diagnosis and opens avenues for targeted psychological and rehabilitative strategies, helping to prevent chronicity and supporting functional recovery.

## Figures and Tables

**Table 1:** Comparison of PCS and FND/FCD clinical and diagnostic features.

Domain	PCS	FND/FCD
Etiology	Biomechanical Brain Trauma (e.g., mTBI)	Functional Brain Network Dysfunction
Symptoms	Headache, dizziness, fatigue, cognitive complaints, mood changes	Motor/sensory symptoms, PNES, cognitive complaints, dissociation
Objective Findings	Often normal imaging; subtle DTI/fMRI anomalies in some cases	Typically normal imaging; positive functional signs on exam
Diagnostic Criteria	Based on symptom constellation post-mTBI; ICD-10/DSM-IV (historical)	Positive signs (DSM-5/ICD-11); symptom incongruence with disease patterns
Neuropsychological Profile	May show subtle deficits; often normal in chronic phase	Discrepancy between complaints and test performance; variable consistency
Biomarkers	GFAP, NF-L (acute phase); exploratory use of miRNAs, inflammatory markers	No established biomarkers; cortisol explored as a state/trait indicator

**Table 2:** Shared and distinct risk factors in PCS and FND/FCD.

Risk Factor	PCS	FND/FCD	Shared?
Female sex Prior psychiatric illness Personality traits	High prevalence	High prevalence	✔
	Depression, anxiety	Depression, anxiety, PTSD	✔
	High neuroticism, somatic anxiety	Neuroticism, perfectionism, alexithymia	✔
Trauma history Multiple prior concussions	Psychological and physical trauma	Early life adversity, abuse	✔
	Associated with chronic symptoms	Less directly implicated	Unclear; under-investigated
Positive clinical signs Diagnostic delay Neuroimaging changes Risk Factor	Typically absent	Hoover’s sign, distractibility, variability	✖
	Often overlooked in chronic phase	Frequently misdiagnosed	✔
	White matter disruption, network dysfunction	Network dysfunction in emotion/agency networks	✔
	PCS	FND/FCD	Shared?
Female sex Prior psychiatric illness	High prevalence	High prevalence	✔
	Depression, anxiety	Depression, anxiety, PTSD	✔

Footnote to Table 2: Although prior concussions are strongly associated with PCS, their role in FND remains insufficiently studied. Further investigation is needed to determine whether cumulative minor injuries influence functional symptom emergence.

**Table 3:** Comparative summary of biomarker timing and specificity in PCS vs. FND.

Biomarker	Acute PCS	Chronic PCS	FND	Notes
GFAP	+(within 24 h)	±(weeks to months)	-	May persist in some chronic cases
NF-L	+(24–72 h)	±	-	Elevated in axonal injury; low specificity for symptoms
Cortisol	±	±	±	May reflect stress/reactivity in both conditions
MicroRNAs	±	Research-phase	Unknown	Promising direction

## Glossary

PCS	Post-Concussion Syndrome
FND	Functional Neurological Disorder
FCD	Functional Cognitive Disorder
TBI	Traumatic Brain Injury
GFAP	Glial Fibrillary Acidic Protein
NF-L	Neurofilament Light Chain
